# One Reason Why so Many Marriages Are Unhappy

**Published:** 1913-07

**Authors:** 


### One Reason Why So Many Marriages Are Unhappy.

In an article entitled "A Girl and Her Future," in the April *Woman's Home Companion,* the author says in part:

"One does not wonder so much that there are many unhappy marriages; it is amazing, rather, that there are not more, considering how little most of us know about marriage and how little we prepare for it. Yet it is certain that if in our schools and colleges—if especially in our homes—girls were encouraged and trained to think and speak frankly of the duties of wifehood and motherhood that are likely to come to them; if it were admitted to be a mark of unintelligence not to prepare for these duties, the rate of happy marriages would be likely to increase enormously.

"If I were asked for practical suggestions I would say, begin at the beginning. Learn how to do every kind of practical work that is or ever will be required in the average home. Learn every part of a household's management as an engineer learns to know every part of his machine. You may think it a trifle to learn to dust carefully and well, to know how to cook a tasty meal, how to select meat and how to buy judiciously, to prepare tempting invalid dishes, to nurse a little child who is ill, or to entertain one who is convalescent, to make clothes, to write proper notes, to judge between good and bad in furniture, pictures and house furnishings. These things are all allied with the success or failure of your future life."—*Southern Clinic.*

